# Prooxidant-Antioxidant Balance in Patients with Systemic Lupus Erythematosus and Its Relationship with Clinical and Laboratory Findings

**DOI:** 10.1155/2016/4343514

**Published:** 2016-01-26

**Authors:** Seyyed Mehdi Jafari, Saeedeh Salimi, Alireza Nakhaee, Hamed Kalani, Shima Tavallaie, Farzaneh Farajian-Mashhadi, Zahra Zakeri, Mahnaz Sandoughi

**Affiliations:** ^1^Department of Clinical Biochemistry, School of Medicine, Zahedan University of Medical Sciences, Zahedan 9816743175, Iran; ^2^Cellular and Molecular Research Center, Zahedan University of Medical Sciences, Zahedan 9816743175, Iran; ^3^Department of Parasitology and Mycology, School of Medicine, Isfahan University of Medical Sciences, Isfahan, Iran; ^4^Department of Nutrition and Biochemistry, Faculty of Medicine, Mashhad University of Medical Sciences, Mashhad, Iran; ^5^Department of Pharmacology, School of Medicine, Zahedan University of Medical Sciences, Zahedan 9816743175, Iran; ^6^Department of Internal Medicine, School of Medicine, Zahedan University of Medical Sciences, Zahedan 9816743175, Iran

## Abstract

*Aim*. This study was aimed at evaluating prooxidant-antioxidant balance (PAB) in patients with systemic lupus erythematosus (SLE) and its relationship with laboratory findings and clinical manifestations.* Methods*. In this case-control study, 60 patients with SLE and 60 healthy individuals were enrolled. The blood samples were collected and their sera were separated. Subsequently, the prooxidant-antioxidant balance value was evaluated using PAB assay for each sample.* Results*. The mean of PAB values in SLE patients was significantly higher than healthy controls (147.3 ± 42 versus 84.8 ± 32.2 HK, *P* < 0.0001). Furthermore, in SLE patients, there was a positive significant correlation between the PAB and erythrocyte sedimentation rate (ESR) (*r* = 0.492, *P* < 0.001). In addition, the PAB values in patients with alopecia, discoid rash, oral ulcers, arthritis, and nephritis were significantly higher than those without these manifestations.* Conclusion*. The findings of current study showed that the mean of PAB was significantly higher in SLE patients and PAB was correlated with ESR. Moreover increased PAB was found in SLE patients with alopecia, discoid rash, oral ulcers, arthritis, and nephritis. These findings suggest that the measurement of PAB may be useful to show oxidative stress condition in SLE patients.

## 1. Introduction

Systemic lupus erythematosus (SLE) is an autoimmune inflammatory disease which can involve nearly all the body's tissues and organs such as kidneys, skin, cardiovascular system, lungs, joints, muscles, and nervous system [1]. SLE is characterized by a broad spectrum of clinical presentation and the existence of multiple autoantibodies, mostly involving women in reproductive age [2, 3]. The course of the disease is variable with periods of remission and relapse [4]. Despite the fact that important advances in understanding its etiopathogenesis have been made over the last several years, the exact cause of lupus is unknown [4]. The environmental factors and genetic and hormonal disorders are common factors that contribute to development of this disease [5]. In recent years, it has been demonstrated that free radical damage and oxidative stress play a major role in pathogenesis and clinical symptoms in patients with SLE [6]. Free radicals and reactive oxygen species (ROS) are generated constantly as part of cellular metabolic processes. These free radicals are deactivated by a chain reaction-breaking antioxidant defense system consisting of enzymes and numerous nonenzymatic antioxidants [7]. Oxidative stress is essentially an imbalance between productions of reactive oxygen species (ROS) and antioxidant defense mechanism [8]. The increased production of free radicals disrupts redox status and can increase the expression of a wide variety of inflammatory molecules that may be important causes of the inflammation and tissue damage [9]. Several studies suggested that oxidative stress could be a risk factor for autoimmune diseases such as SLE [8–10]. Impaired antioxidant status has been reported in the saliva of SLE patients too [11].

The oxidative stress is evaluated by various methods in which oxidants and antioxidants are measured separately [12–14]. Accordingly, the PAB (prooxidant-antioxidant balance) method was developed to provide a rapid and reliable detection of oxidant and antioxidant balance simultaneously [15]. To the best of our knowledge there is no published report about the PAB assay in SLE patients; therefore current study was aimed at evaluating prooxidant-antioxidant balance in patients with SLE and its relationship with laboratory findings and clinical manifestations.

## 2. Materials and Methods

### 2.1. Participants

This study was confirmed by the University Research Ethics Committee (UREC) of the Zahedan University of Medical Science. In this case-control study, 60 patients with SLE (54 women and 6 men) with a mean age of 30.57 ± 7.74 years as a test group and 60 healthy individuals (54 women and 6 men) with a mean age of 31.07 ± 7.5 years as a control group were enrolled. All participants completed the informed consent form. Patients in this study were selected in accordance with the American College of Rheumatology (ACR) criteria for SLE. Moreover, the participants in the control group had no systemic diseases and their antinuclear antibody (ANA) test was negative.

### 2.2. Sampling

Two-milliliter fasting blood samples were collected from all the subjects. The blood samples were centrifuged immediately and their sera were separated and then stored at −80°C until use.

### 2.3. Chemicals

3,3′,5,5′-Tetramethylbenzidine (TMB) powder and chloramine T trihydrate were obtained from AppliChem (Darmstadt, Germany), peroxidase enzyme and uric acid were obtained from Sigma-Aldrich (St. Louis, MO), and hydrogen peroxide and DMSO were obtained from Merck Company (Darmstadt, Germany).

### 2.4. Prooxidant-Antioxidant Balance (PAB) Assay

The PAB assay was performed in accordance with the method explained in detail by Alamdari et al. [16]. Briefly, the* PAB* method was used to determine the* prooxidant-antioxidant balance* value.

#### 2.4.1. Preparation of Solutions

60 mg TMB powder was weighed and 10 mL DMSO was added. The TMB cation was prepared as follows: 20 mL of acetate buffer [0.05 M buffer, pH 4.5], 400 *μ*L of TMB/DMSO, and 70 *μ*L of fresh chloramine T (100 mM) solution were mixed and incubated at room temperature for 2 hours in dark place; 25 U of peroxidase enzyme solution was added into 20 mL TMB cation, dispensed into 1 mL aliquots, and frozen at −20°C; the TMB solution was prepared with 200 *μ*L of TMB/DMSO in 10 mL of acetate buffer [0.05 M buffer, pH = 5.8]; in order to prepare working solution 1 mL TMB cation was added in 10 mL of TMB solution, incubated at room temperature for 2 min in a dark place, and used freshly.

#### 2.4.2. Procedure

10 *μ*L of 200 *μ*L of working solution, in each well of a 96-well plate, each sample, standard or blank, was added, and the mixture was allowed to incubate at 37°C for 12 min in a dark place; at the end of the incubation time, 100 *μ*L of 2 N HCl was added to each well; the absorbance at 450 nm was read against distilled water. Hydrogen peroxide was considered as a standard in different concentrations (0–100%). After drawing the standard curve, the PAB value was calculated for each unknown sample according to the standard curve using OD obtained for each sample in the test and control groups.

### 2.5. Data Analysis

All the statistical analysis was carried out by SPSS software version 16. Data have been presented as mean ± SD as well as percentage. Data in each group showed a normal distribution by Kolmogorov-Smirnov (K-S) statistical test. Comparison between test and control groups was performed using the independent sample *t*-test. In addition, Pearson's correlation coefficient test was used to determine the correlation among different factors.

## 3. Results

Table 1 shows demographic and laboratory data related to the patients and control groups. The data analysis showed that there was statistically a significant difference between means of RBC, hemoglobin, WBC, platelets, hematocrit, and ESR in two independent groups.

**Figure 1:**
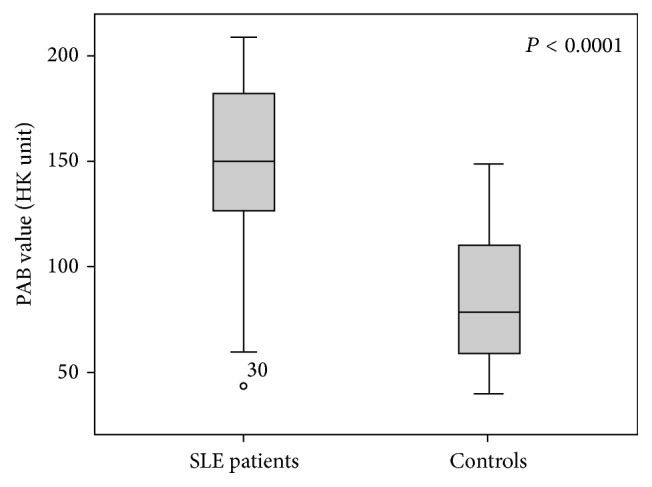
Comparison of PAB values between SLE patients and control group.

As shown in Figure 1, the mean of PAB values in SLE patients (147.3 ± 42 HK) was higher than the healthy group (84.8 ± 32.2 HK) and it was highly significant statistically (*P* < 0.0001).

In addition, Table 2 shows the correlation between the PAB value and laboratory findings in the patients and control groups. In SLE patients, there was a positive significant correlation between the PAB value and erythrocyte sedimentation rate (ESR) (*r* = 0.492, *P* < 0.001).

Moreover as has been shown in Table 3 the PAB values were significantly different in SLE patients with and without some clinical manifestations, such as alopecia (164 ± 42 versus 132 ± 36, *P* = 0.002), discoid rash (176 ± 42 versus 140 ± 39, *P* = 0.005), oral ulcers (158 ± 42 versus 132 ± 38, *P* = 0.002), arthritis (158 ± 39 versus 112 ± 29, *P* = 0.0001), and nephritis (182 ± 29 versus 134 ± 40, *P* = 0.0002). There were no correlations between PAB values and photosensitivity, malar rash, anemia, serositis, neurological involvement, ANA, and anti-dsDNA manifestations.

## 4. Discussion

Systemic lupus erythematosus is an autoimmune disorder that affects many different tissues and organs in the body [1]. The exact cause of the disease is not fully known but environmental factors and genetic and hormonal disorders are involved in development of SLE [4]. Some studies revealed that the inability of the antioxidant defense system to cope with oxidative stress plays an important role in the pathogenesis of SLE [17, 18]. The increased oxidative stress in patients with SLE can lead to the oxidative modifications of proteins, lipids, and DNA in cells [19]. The main objective of the present study was therefore to evaluate prooxidant-antioxidant balance (PAB) in patients with systemic lupus erythematosus (SLE) and its relationship with laboratory findings and clinical manifestations. In this study, we showed that an increase in PAB value among SLE patients in comparison to the control group and PAB was correlated with ESR. Moreover increased PAB was found in SLE patients with alopecia, discoid rash, oral ulcers, arthritis, and nephritis. Zhang et al. [20] and Morgan et al. [21] demonstrated that the protein oxidation markers are associated with chronic injuries in SLE patients. These studies support strongly the role of oxidative stress in pathogenesis of SLE. In addition Shah et al. [22] showed that malondialdehyde level as a marker of lipid peroxidation was significantly higher in SLE patients compared to control group. In a recent study, the antioxidant activities of some enzymes, namely, superoxide dismutase, catalase, and glutathione peroxidase, and the level of glutathione molecules were significantly lower in SLE patients than in controls. Likewise, in another study authors revealed a significant relationship between malondialdehyde level and ESR in patients with SLE [23]. Similarly the present study showed a significant positive correlation between the PAB value and ESR in SLE patients. In a study, urinary F2-isoprostane excretion was evaluated as a biomarker for oxidative stress, and the findings showed that there was no significant correlation between this biomarker and some major symptoms such as inflammation [24]. Researchers elsewhere illustrated that antibody levels against hydroxynonenal and malondialdehyde in sera obtained from SLE patients had a significant positive correlation with disease activity, so that the patients with score higher than 6, determined by SLE disease activity index (SLEDAI), had higher levels of the mentioned antibodies [25]. However, it appears that patients with inactive disease also have high levels of lipoperoxidation and protein oxidation [26]. Furthermore, it was revealed that the balance between oxidants and antioxidants is impaired in lupus patients and the consumption of antioxidants such as vitamin E, eicosapentaenoic acid, and docosahexaenoic acid can be effective in reducing the symptoms of the disease [27]. It was also observed that the use of vitamins C and E leads to a considerable decrease in lipid peroxidation, but not in other evaluated oxidative stress markers [28]. In the current study, the PAB value evaluation showed that the prooxidant-antioxidant balance in SLE patients was significantly higher than in healthy controls. Therefore, the findings of this study showed that the PAB method can be used as a new method representing the intensity of oxidative stress in patients with SLE. Accordingly, at the final point, we suggest strongly that antioxidants, as a complementary therapy, should be always added to medications of SLE patients. In conclusion, increased PAB values were observed in SLE patients compared to controls. Higher PAB was found in SLE patients with alopecia. In addition PAB was correlated with ESR. These findings suggest that the measurement of PAB may be useful to show oxidative stress condition in SLE patients.

## Figures and Tables

**Table 1:** Demographic and laboratory characteristics of SLE patients and healthy controls.

Parameters	SLE patients *n* = 60	Healthy controls *n* = 60	*P* value
Sex (M/F)	54/6	53/7	1
Age (years)	30.6 ± 7.8	31.1 ± 7.5	0.7
Hemoglobin (g/dL)	11.1 ± 1	12.7 ± 1.7	<0.0001
RBC (×1.000000/mL)	4.3 ± 0.6	4.7 ± 0.64	0.0002
WBC (×1.000/mL)	7.1 ± 2.3	7.3 ± 1.8	0.6
Platelets (×1.000/mL)	199.6 ± 67.4	223.8 ± 48.7	0.03
Hematocrit (%)	34.1 ± 2.8	37.8 ± 4.7	<0.0001
ESR (mm/h)	36.1 ± 15.9	18.6 ± 7.3	<0.0001
PAB (HK unit)	147.3 ± 42	84.8 ± 32.2	<0.001

F: female; M: male; Hb: hemoglobin; RBC: red blood cells; WBC: white blood cells; ESR: erythrocyte sedimentation rate.

**Table 2:** Correlation of PAB values and laboratory findings in patients with SLE and healthy controls.

Parameters	PAB (HK unit)			
	SLE patients		Healthy controls	
	*R* ^2^	*P* value	*R* ^2^	*P* value
Hemoglobin (g/dL)	−0.2	0.1	−0.2	0.2
RBC (×1.000000/mL)	−0.1	0.5	−0.1	0.5
WBC (×1.000/mL)	−0.1	0.4	0.06	0.7
Platelets (×1.000/mL)	0.08	0.5	0.04	0.8
Hematocrit (%)	−0.1	0.4	−0.14	0.3
ESR (mm/h)	0.5	<0.0001	0.2	0.09

**Table 3:** Comparison of PAB values between SLE patients with and without different manifestation.

SLE manifestations	PAB (UK units)		*P* value
	SLE patients with	SLE patients without	
Photosensitivity	152 ± 37	140 ± 49	0.3
Alopecia	164 ± 42	132 ± 36	0.002
Malar rash	154 ± 46	140 ± 33	0.1
Discoid rash	176 ± 42	140 ± 39	0.005
Oral ulcers	158 ± 42	132 ± 38	002
Anemia	150 ± 38	144 ± 47	0.6
Arthritis	158 ± 39	112 ± 29	0.0001
Nephritis	182 ± 29	134 ± 40	0.0002
Serositis	154 ± 58	147 ± 41	0.7
Neurological involvement	151 ± 36	146 ± 44	0.7
ANA	147 ± 42	147 ± 48	1
Anti-dsDNA	144 ± 44	154 ± 38	0.4
